# The CAM-P-OS study protocol: a prospective randomized multicenter trial evaluating an active controlled motion device in the rehabilitation of surgically treated, isolated ankle fractures of Weber types B and C

**DOI:** 10.1186/s13063-025-09169-y

**Published:** 2025-10-06

**Authors:** Michael Nienhaus, Kai Kronfeld, Olga Schulowski, Claudia Wolf, Juergen Konradi, Ulrich Betz, Erol Gercek

**Affiliations:** 1 Department of Orthopedics and Traumatology, University Medical Center of the Johannes Gutenberg University, Mainz, Germany; 2 Interdisciplinary Center for Clinical Trials, University Medical Center of the Johannes Gutenberg University, Mainz, Germany; 3 Institute of Physical Therapy, Prevention and Rehabilitation, University Medical Center of the Johannes Gutenberg University, Mainz, Germany

**Keywords:** Ankle fractures, Rehabilitation, Active-controlled motion, Trial protocol, Rct, Medical device

## Abstract

**Background:**

Displaced ankle fractures classified as Weber types B and C are common across all age groups. The standard treatment involves open reduction and internal fixation, followed by a rehabilitation program that includes 6 weeks of partial weightbearing. During this period, both passive and active range-of-motion exercises are performed under the supervision of a physiotherapist. Recently, a new device has become available, allowing active controlled motion of the ankle joint at home on top of the normal rehabilitation protocol. The purpose of this controlled randomized trial was to evaluate the superiority of this device as a supplement in standard rehabilitation protocols after distal fibular fractures.

**Methods:**

This prospective controlled multicenter trial will include a minimum of 58 patients who have undergone surgical treatment for isolated distal fibular fractures of Weber types B and C. These patients will be randomized into 2 groups: Group 1 will undergo a standardized rehabilitation protocol with active and passive physiotherapy under partial weightbearing for 6 weeks with the active controlled motion device at home on top. Group 2 will receive the same rehabilitation without the device. Three German hospitals with different levels of care and 6 rehabilitation centers are involved in this study. The estimated study duration of 12 months started in October 2024. The follow-up will last 6 months after the recruitment of the last patient. The following assessments are performed: baseline after surgery; after 6 weeks; at 3 and 6 months; and with check-in calls after 2 and 4 weeks. The evaluation of effectiveness is based on FAOS, NRS, and SF-36 scores; changes in employment during the study; return-to-work; return-to-sports; and time-to-unrestricted weightbearing.

**Discussion:**

This trial aims to collect valid data to determine whether the use of an active controlled motion device as a supplement to a standardized physiotherapy protocol after surgical treatment of ankle fractures leads to significantly improved outcomes. After evaluation of the results by the German Joint Federal Committee, a final assessment is made to make this device part of a future treatment protocol in the aftercare of surgically treated distal fibular fractures of Weber types B and C.

**Trial registration:**

The trial is registered in the International Clinical Trials Registry Platform of the World Health Organization with the Main-ID DRKS00034202 on July 22nd, 2024 (https://trialsearch.who.int/Trial2.aspx?TrialID=DRKS00034202).

## Administrative information

Note: the numbers in curly brackets in this protocol refer to SPIRIT checklist item numbers. The order of the items has been modified to group similar items (see http://www.equator-network.org/reporting-guidelines/spirit-2013-statement-defining-standard-protocol-items-for-clinical-trials/).

**Table:** 

Title {1}	The CAM-P-OS study protocol: a prospective randomized multicenter trial evaluating an active controlled motion device in the rehabilitation of surgically treated, isolated ankle fractures Weber Type B and C.
Trial registration {2a and 2b}.	International Clinical Trials Registry Platform of the World Health Organization (https://trialsearch.who.int/Trial2.aspx?TrialID=DRKS00034202). Registered July 22nd, 2024.
Protocol version {3}	Version 1.1, June 11th, 2024
Funding {4}	German Joint Federal Committee; Berlin/Germany (G-BA Gemeinsamer Bundesausschuss Berlin), the highest decision-making body for joint self-administration in the German healthcare system.
Author details {5a}	^1^ Department of Orthopedics and Traumatology, University Medical Center of the Johannes Gutenberg University, Mainz, Germany. ^2^ Interdisciplinary Center for Clinical Trials, University Medical Center of the Johannes Gutenberg University, Mainz, Germany. ^3^ Institute of Physical Therapy, Prevention and Rehabilitation, University Medical Center of the Johannes Gutenberg University, Mainz, Germany
Name and contact information for the trial sponsor {5b}	University Medical Center of the Johannes Gutenberg University, Mainz, Germany. Langenbeckstraße 1, 55131 Mainz, Germany
Role of sponsor {5c}	The sponsor developed the study design, specified what data was to be collected and organized data collection, evaluation and interpretation.

## Introduction

### Background and rationale {6a}

Fractures of the lower limb, including the ankle joint, are common, with an incidence of 145/100,000 inhabitants. In 2021, 120,320 patients with the ICD-10 code S82 were treated in German hospitals [1]. The distal fibular fracture type Weber B is the most common fracture type [2]. The standard treatment for significantly displaced Weber type B or C fractures is the combination of interfragmentary screw compression with neutralization plating (locking or nonlocking) with or without a set screw, depending on syndesmotic injury. Adequate postoperative aftercare is controversial in the current literature [3, 4]. After surgery, early rehabilitation with partial weightbearing and exercise under physiotherapist supervision seems to lower the risk of deep vein thrombosis, edema and pain and helps patients recover a full range of movement and normal proprioception [5].

A supporting active controlled motion (ACM) device with continuous active motion (CAM) could already show encouraging significant benefits to knee joint proprioception in the early postoperative phase after anterior cruciate ligament repair [6]. This device combines exercises with muscular activity in a closed kinetic chain via normal proprioceptive mechanisms as part of the neuromuscular training required by Rivera in 1994 [7]. The usage of this apparatus in the aftercare of surgically treated distal fibular fractures led to earlier full weight bearing, better functional scores (e.g., VAS and AOFAS), and an earlier return to work [8].

Our hypothesis is that this ACM device optimizes the actual treatment after ankle fracture and represents a potential addition to standard physiotherapy. The German Joint Federal Committee, Berlin (G-BA), and our department initiated the CAM-P-OS trial (Continuous Active Motion versus Physiotherapy in “Obere *S*prunggelenkfrakturen” (=ankle fractures)) to gain the knowledge necessary for the final assessment of the benefits to make this splint part of a future treatment.

### Objectives {7}

The aim of the CAM-P-OS study is to evaluate if the addition of an ACM device to standard physiotherapy after surgically treated distal fibular fractures Type Weber B and C is safe, effective and leads to superior outcomes in comparison to standard physiotherapy alone.

The procedures to achieve the objectives are the following:

- Recruitment of a minimum of 58 eligible patients with isolated distal fibular fractures of Weber types B and C and indications for open reduction and internal fixation (ORIF).
- The initial assessment includes the *F*oot and *A*nkle *O*utcome *S*core (FAOS) [9–11], *N*umeric *R*ating *S*cale (NRS) [12], *S*hort-*F*orm-36 questionnaire (SF-36) [13], *r*ange-*o*f-*m*ovement (ROM), preexisting and accompanying illnesses, preliminary and accompanying therapies, type of job prior to ankle injury, wound conditions and joint-related swelling at baseline for recruited patients.
- Patients are randomly assigned to the intervention group (standard physiotherapy protocol with ACM device) or the control group (standard physiotherapy protocol alone).
- Monitoring of therapy adherence is measured with a self-reported therapy diary and check-in calls 2 and 4 weeks after surgery.
- Repetition of initial assessments 6 weeks as well as 3 and 6 months after surgery.
- Comparison of the primary and secondary endpoints between the two groups.

### Trial design {8}

The CAM-P-OS trial is a prospective, investigator-blinded, randomized and controlled comparative study with a parallel group design. The aim of this study is to evaluate whether the addition of an active controlled motion device in the rehabilitation of patients with surgically treated, isolated distal fibular fractures leads to superior results in terms of joint function.

## Methods: participants, interventions and outcomes

### Study setting {9}

The CAM-P-OS trial will be performed at a Level 1 trauma center (Department of Orthopedics and Traumatology, University Medical Center of the Johannes Gutenberg University, Mainz, Germany) and two adjacent local trauma centers and academic teaching hospitals of the University Medical Center of the Johannes Gutenberg University. Patients are recruited immediately after trauma in the emergency room, otherwise in the ward after surgery. If necessary, the internal digital documenting system of the clinics can also be used for screening.

### Eligibility criteria {10}

Inclusion: Participants are eligible for the study if they meet the following inclusion criteria:

1. Surgically treated, isolated fracture of the distal fibula Weber Type B and C with or without syndesmotic injury
2. Age ≥ 18 years
3. Stable osteosynthesis for partial weightbearing and active/passive movement
4. FAOS-ADL ≤ 80 at the baseline visit
5. Signed, informed consent

Exclusion: If participants meet the following criteria prior to or at the first visit after surgery, they are not eligible for trial participation:

1. Comorbidities
  - Cognitive impairment, e.g., dementia
  - Osteoporosis (with specific medication, e.g., bisphosphonates)
  - Rheumatic diseases
  - Peripheral artery disease (Fontaine Grade IV/Rutherford 3/5 + 3/6)
  - Walking-limiting diseases (e.g., amputation, neurologic diseases)
2. Trauma-related comorbidities
  - Polytrauma
  - Severe brain injury
  - Open fractures
  - Involvement of the medial malleolus
  - Combined fractures of the ipsilateral foot
3. Pregnancy
4. Lack of German to understand the protocol or to sign informed consent.

### Who will take informed consent? {26a}

After radiologic diagnosis of an isolated ankle fracture type Weber B or C in the emergency room, patients were evaluated to determine whether they met the inclusion criteria prior to surgery. During an operation preparation appointment in the outpatient department, the participants are interviewed by an investigator and a study nurse and will receive initial study information. After a period of reflection and the possibility of having questions answered, the patient´s written consent is obtained and confirmed by the investigator.

### Additional consent provisions for collection and use of participant data and biological specimens {26b}

N/a. With this trial, no additional collection or use of participant data or biological specimens is planned in ancillary studies.

## Interventions

### Intervention description {11a}

**Fig. 1:**
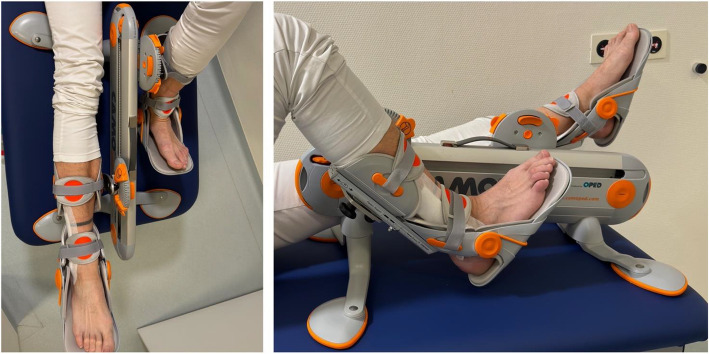
Mounted ACM device during exercise (Camoped©, OPED® GmbH, Valley, Germany)

After surgery, patients with a distal fibular fracture receive physiotherapy with partial weightbearing (20 kg) and intensive active and passive mobilization of the injured ankle joint during the 1–2 days of hospital stay. After discharge from the clinic, 12 guaranteed units of outpatient physiotherapy follow with 2 appointments a week in one of the cooperating rehabilitation centers. Each physiotherapy session lasts a minimum of 20 min [14]. Additionally, a self-exercise program that includes a daily exercise time of at least 2 × 20 min per day is developed by physiotherapists with respect to participants’ conditions and should be completed by the patients at home. This rehabilitation protocol will then be expanded by an ACM device for 8 weeks that should be used for 20 min 2 times per day. The ACM-device (e.g., Camoped©, OPED® GmbH, Valley, Germany) used serves mobilization of the hip, knee and ankle joints [6, 8]. The movement of the injured leg occurs through the active extension and flexion of the contralateral, uninjured leg. This device is delivered to the patient’s home by a medical device advisor from the manufacturer. The technical briefing will be carried out by these trained employees and then applied independently by the patient. For application, the user sits/lies in front of the ACM device and places both legs on the pedals provided. The soles of the feet should be in full contact with the foot plate, and the lower leg shell should be individually adjusted to the lower leg length. After correct positioning and adjustments, repetitive movement exercises can be started (Fig. 1). For further product information contact, https://www.youtube.com/watch?v=uR8el9Pq0xo (instructions for use only available in German).

### Explanation for the choice of comparators {6b}

The control group receives the same postoperative rehabilitation protocol without the ACM device.

### Criteria for discontinuing or modifying allocated interventions {11b}

Throughout the trial, every participant in the intervention group can adjust their weightbearing to the pedals to the individual pain level after surgery. In the beginning, it is possible to move the injured ankle passively and indirectly through the uninjured leg. During further treatment, partial weightbearing with active/assisted CAM can be achieved stepwise.

Trial withdrawal by the study participants is possible at any time without giving reasons or any resulting disadvantages. A representation for the visits remains possible. No further data will be collected, although existing data will remain in the database. Nevertheless, an attempt is made to carry out a final visit to obtain final test results.

In addition, study participants may be excluded from the clinical study for the following reasons:

- At the request of the sponsor when stopping the entire study prematurely or for other regulatory reasons
- Newly discovered risks for study participants
- Proof of product ineffectiveness
- Occurrence of, until then, completely unknown AEs or known AEs with unknown frequencies
- Medical or ethical reasons that adversely affect the further conduct of clinical studies

Early drop-out participants will not be replaced.

### Strategies to improve adherence to interventions {11c}

In this trial, several strategies for improving adherence are implemented. During the 8 weeks of rehabilitation, the participants are required to document the actual daily training times in a training diary, which should be returned to the study centers afterwards. Furthermore, the physiotherapists involved should inquire about adherence to participants’ own home treatments, assess patients’ compliance and therapy adherence twice via a questionnaire and send the results to the test centers. In addition, check-in calls 2 and 4 weeks after surgery should help prevent any difficulties, ensure and increase compliance to improve data quality and validity and keep the drop-out rate low.

### Relevant concomitant care permitted or prohibited during the trial {11d}

The study participants receive standardized physiotherapy as described above. If necessary, an orthosis will be prescribed, as well as crutches during partial weight bearing. All aids will be documented in the electronic case report form (eCRF).

### Provisions for post-trial care {30}

The sponsor (or a designated representative of the sponsor) has an insurance taken out. The general insurance conditions are provided to the participants and investigators. Any health impairment that may have occurred because of participation in the study must be reported to the insurance company immediately, if needed, after consultation with the investigator.

### Outcomes {12}

The primary endpoint is improvement in joint function 3 months after surgery, measured with the FAOS-ADL, a subscore of 17 items that are well validated and available in the German language Foot and Ankle Outcome Score (FAOS) [9–11]. It consists of 42 questions in 5 patient-relevant chapters: (1) pain (9 items); (2) other symptoms, e.g., swelling (7 items); (3) Activities of Daily Living (ADL, 17 items); (4) sport and recreational activities (5 items); (5) Foot- and Ankle-Related Quality of Life (4 items).

Various parameters were chosen as secondary endpoints to draw conclusions about the success of ACM therapy or the study process (see Table 1 for the time schedule):

- Increase in activities of daily living, measured with the FAOS-ADL
- Reduction in fracture-/surgery-related symptoms measured with the FAOS subscore “Other symptoms”
- Pain relief, measured with the FAOS subscore “Pain” and the NRS [12]
- Increase in sports and recreational activities, measured with the FAOS subscore “Sport and Recreational activities”
- Increase in quality of life, measured with the FAOS subscore “Foot- and Ankle-Related Quality of Life”
- Increase in health-related quality of life, measured with the Short-Form-36 questionnaire (SF-36) [13, 15]
- Type of job prior to ankle injury and after rehabilitation (“none,” “job with physical activity,” “job without physical activity,” “job with both physical and nonphysical activities”)
- Time to “Return-to-work”
- Time to full weightbearing
- Time to “return to sports,” which means the time to the individual sport level prior to injury.
- Changes in oral pain medication
- Therapy adherence, measured with a self-reported therapy diary
- Range of motion, measured with a goniometer
- Presence of swelling, measured with a measuring tape around the ankle joint, the thinnest diameter of the lower limb and the foot at the level of the navicular bone
- Wound evaluation by an observer: “normal,” “delayed” (> 14 days after closure), and “infected” (with indication for revision surgery) at baseline, after 6 weeks and after 3 and 6 months
- Wound evaluation by patients: “normal/inconspicuous,” “severely reddened,” “severely oozing,” and “other” during check-in calls 2 and 4 weeks after surgery
- Occurrence of adverse events (AEs) and revision surgery in terms of frequency and severity (query); in the case of setscrew-removal, no AE is documented
- Occurrence of patient withdrawal due to adverse events (query)

**Table 1:** Participant timeline

Action	Visits						
	Screening	Visit1 (Baseline Visit)	Visit 2 (by telephone)	Visit 3 (by telephone)	Visit 4	Visit 5	Visit 6
Trial day	Accident day to −1 day	2 days ± 1 post-op (day 0)	In 2nd week post-op	In 4th week post-op	6 weeks ± 7 days post-op	3 months (90 ± 7 days) after baseline	6 months (180 ± 14 days) after baseline
Patient information and informed consent	✖	✖					
Inclusion/exclusion criteria	✖ (3, 5)^a^ ✖^b^	✖ (1, 2, 4) ✖^b^					
Demographics (e.g. sex, age, height, weight, BMI)	✖^b^	✖^b^					
Type of employment	✖^b^	✖^b^	✖	✖	✖	✖	✖
Date of return to work			✖	✖	✖	✖	✖
Previous diseases	✖^b^	✖^b^					
Concomitant diseases	✖^b^	✖^b^	✖	✖	✖	✖	✖
Previous treatments	✖^b^	✖^b^					
Previous and concomitant treatments (incl. medication)	✖^b^	✖^b^	✖	✖	✖	✖	✖
Request therapy process (appointment allocation, problems with splint, etc.)			✖	✖			
FAOS		✖			✖	✖	✖
SF-36		✖			✖	✖	✖
ROM		✖			✖	✖	✖
Condition of the wound		✖	✖^c^	✖^c^	✖	✖	✖
Joint-related edema		✖			✖	✖	✖
NRS		✖	✖	✖	✖	✖	✖
Randomization		✖					
Handing out the training diary		✖					
Handing out a prescription for physiotherapy		✖					
Handing out the Information for physiotherapy		✖					
If necessary, hand out prescription for splint		✖					
Training of the study product		(✖)^d^					
Checking the individual training time (handover of training diary)						✖	
Physiotherapist's estimate of therapy adherence				✖	✖		
Adverse events			✖	✖	✖	✖	✖
Date of full weight bearing (X-ray check)					✖	✖	
Date of return to sports				✖	✖	✖	✖
End of trial (final visit)							✖

^a^Inclusion criteria 1, 2 and 4 can be recorded only postoperatively, but points 3 and 5 can be recorded beforehand, but they are assigned to the baseline visit in the eCRF and documented there

^b^These data can already be collected at Visit 0 screening but are assigned to the baseline visit in the eCRF and documented there

^c^Patient self-reports; therefore, this value differs from that of the medical assessment

^d^This does not take place at the time of visit 1 but between visits 1 and 2

### Participant timeline {13}

Table 1 shows the participant timeline.

### Sample size {14}

**Fig. 2:**
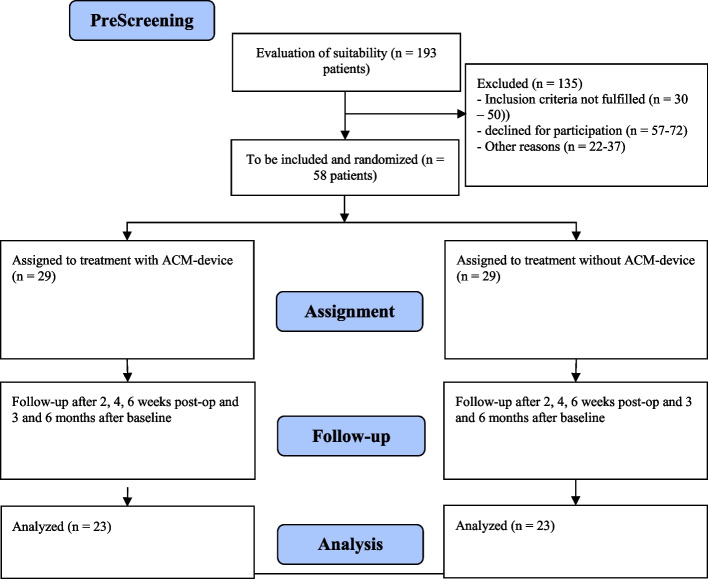
CONSORT flowchart showing the expected number of cases. ACM: active controlled motion, OP: surgery

Currently, no data are available comparing the ACM device with the standard treatment using the FAOS-ADL. Jansen et al. [8] reported a difference of 16.32 points between the two interventions after 3 months in the visual analog scale by Richter et al. [16]. A pooled standard deviation of 15,10 units results in a standardized effect size of *g* = 1.08. With the assumption that the VAS score correlates with the FAOS-ADL score, similar effect sizes to those of the VAS score can be expected. For planning, we assumed that the standardized effect size was somewhat more conservative at *g* = 1.0. According to van den Akker-Scheek [17], van Bergen [11], Desai [18] and Negahban [19], the standard deviation of the FAOS-ADL score is approximately 22 points. Together with the expected standardized effect size of* g* = 1.0, this results in an expected effect of 22 score points in the FAOS-ADL. According to Ackermann [20], the effect can be assessed as clinically relevant since previous studies have shown a minimal clinically relevant difference between 8.3 and 20.0. With a two-sided significance level of 5%, a power of 90% and the assumptions above, 46 patients will be needed in total to test for superiority via a *t*-test (Fig. 2). A dropout rate of 20% is expected so that randomization of 58 patients is necessary. Sample size planning was carried out via SAS, version 9.4 (SAS Institute Inc., Cary, NC, USA).

### Recruitment {15}

The screening to determine the suitability of the 58 patients to be recruited later takes place either in the outpatient departments/emergency rooms of the clinics or in the ward. The clinic's internal documentation system can also be used for screening.

Measures to increase the recruitment rate are as follows:

- Error analysis/discussions between sponsor and test center
- Training of test center employees
- If necessary, monthly evaluation of the recruitment rates
- If a study center is unable to achieve its recruitment goal, missing participants will be recruited by other participating study centers. If this is not possible either, the integration of additional centers will be checked

## Assignment of interventions: allocation

### Sequence generation {16a}

After written informed consent is given, randomization will be performed according to a randomization list with a permuted block, where the block size is predetermined, and center is used as an additional stratification factor. Study patients are divided into groups and randomly assigned to one of two treatment groups within these blocks, with a 1:1 ratio of study patients in each group. Group 1 (intervention) will receive a rehabilitation protocol with a new active controlled motion (ACM) device on top. Group 2 (control) will undergo the same rehabilitation protocol without the ACM-device.

The randomization list will be created by an independent employee of the Interdisciplinary Center for Clinical Trials (IZKS) of the University Medical Center Mainz.

### Concealment mechanism {16b}

The randomization list is stored securely and confidentially at the IZKS of the University Medical Center Mainz and ensures allocation concealment.

### Implementation {16c}

The allocation sequence generation is embedded in the randomization list. The study nurses will enroll participants and randomize them via the randomization list.

## Assignment of interventions: blinding

### Who will be blinded {17a}

The investigators, involved in the performing of the follow-up visits at the respective test centers are blinded throughout the trial. The participants, physiotherapists and study nurses are not blinded. Patients are advised to keep the investigators blinded during visits at 6 weeks and 3 and 6 months after baseline.

### Procedure for unblinding if needed {17b}

After the final follow-up visit of the last recruited patient, the database closure will be performed by the IZKS after a blind data check has been carried out and a statistical analysis plan has been released. The decision on the study end will be made by the principal investigator or the sponsor and the regular unblinding of the treatment allocation will be performed to enable the statistical evaluation of the trial data. An emergency unblinding in the course of the study is not required, since the patients are aware of their treatment allocation.

## Data collection and management

### Plans for assessment and collection of outcomes {18a}

Data are collected at baseline, 6 weeks after surgery, and 3 and 6 months after baseline by blinded investigators with additional check-in calls 2 and 4 weeks after surgery, performed by the study nurses. Data will be entered by site staff in an electronic case record form (eCRF) administered by the IZKS Mainz. Data will be stored at the IZKS Mainz throughout the study. The training diaries of the participants as well as the questionnaires of the physiotherapists will be collected during the visit 3 months after baseline.

### Plans to promote participant retention and complete follow-up {18b}

Participants will start the trial after being provided detailed study information about the possible benefits of a guaranteed rehabilitation protocol directly after discharge from the clinic. Check-in calls should solve any problems with the protocol or the study product and promote participant retention. The patients can withdraw at any time and without giving reasons. Patients who have dropped out are still encouraged to attend the final study visit after 6 months for safety reasons. Data collection takes place during appointments in the outpatient department of the clinics. These appointments are made during the hospital stay and handed out to the patients so that the waiting time for physical and radiological examination and data collection is reduced to a minimum for participants convenience. If a risk of loss to follow-up becomes recognizable, study nurses will contact the concerned patients for completion of the follow-up.

### Data management {19}

Data collection is carried out via an electronic data capture system and is documented in an electronic case report form (eCRF). According to the guidelines of good clinical practice (GCP), the eCRF and relevant printed study files are stored on a secure server or locked room at the University of Mainz for at least 10 years. The study nurses within each clinic will initially receive data entry instructions. Data entry, data changes, and data deletion are documented by the system through an audit trail, stating the relevant study participant, the original and the corrected value, the reason for and the person carrying out the change, including the time of change. Unauthorized access to the data is prevented by the system and software. The database management system can obtain accurate and complete copies of the data in both machine and visually readable forms.

### Confidentiality {27}

At the start of the study, the principal investigator will receive an investigator site file (ISF) containing all essential documents and relevant correspondence. After the study completion the ISF, complete source data and essential documents will be stored in accordance with the legal requirements, especially § 13/10 GCP-V, and the requirements of the ICH-GCP guidelines. The participants receive a Participants Identification Number (PIN) which is a unique code assigned to each individual participating in the study which is documented in a patient identification form and stored in the responsible test center. The documentation of all relevant data is performed in an eCRF.

### Plans for collection, laboratory evaluation and storage of biological specimens for genetic or molecular analysis in this trial/future use {33}

N/a. In this trial no biological samples for genetic or molecular analysis are collected, evaluated or stored.

## Statistical methods

### Statistical methods for primary and secondary outcomes {20a}

The primary estimand (systematic description of the treatment effect) is defined as the absolute change in the FAOS-ADL score after 3 months of therapy compared with baseline. The estimand is made up of five attributes, one of which is handling intercurrent events (ICEs): Intercurrent events are defined as events influencing the interpretability of the observed variables.

Intercurrent event 1: Therapy discontinuation: Treatment policy (the variable is used unchanged regardless of whether the ACM device is used or physiotherapy is not caried out).

Intercurrent event 2: Missing FAOS value after 3 months: Hypothetical (missing values are randomly drawn from the distribution of baseline values of the included patients).

The following hypothesis will be tested: H0: µI = µK vs. H1: µI ≠ µK, where µI and µK are the expected values in the intervention group and the control group, respectively.

The primary estimand is analyzed with a mixed model with fixed factor variables for center, intervention, measurement time (6 weeks, 3 months, or 6 months) and the interaction term of intervention and measurement time. The baseline value before the first intervention is considered a random effect. Compound symmetry is assumed as the covariance structure. The linear contrast at the time of measurement (3 months) is assessed with a *p* value and 95% confidence interval.

A sensitivity analysis is carried out in a similar manner by using ICE1 but not ICE2.

The continuous secondary endpoints are analyzed via similar models as the primary outcome measure. Differences between interventions are presented via estimators from the analysis model and 95% confidence intervals. The dichotomous secondary endpoints will be analyzed via chi-square tests. All the parameters are also evaluated descriptively.

For all effectiveness analyses, the ITT population is the evaluation population.

All hypotheses are tested two-sided at the *α* = 0.05 level.

### Interim analyses {21b}

N/a. No interim analysis is planned.

### Methods for additional analyses (e.g., subgroup analyses) {20b}

N/a. There are no plans to define subgroups for analysis.

### Methods in analysis to handle protocol non-adherence and any statistical methods to handle missing data {20c}

Any deviations from the original statistical plan can be reported in the SAP or in a revised version of the study protocol.

There are no plans to correct missing data.

### Plans to give access to the full protocol, participant-level data and statistical code {31c}

N/a. There are no plans to provide access to the full protocol, participant-level data or statistical code.

## Oversight and monitoring

### Composition of the coordinating center and trial steering committee {5d}

The CAM-P-OS trial is a multicenter study with 3 participating trauma clinics. Study coordination is performed by University Medical Center Mainz. The principal investigator is responsible for trial supervision and participants’ medical questions. Data management is described in a data management plan (DMP) under responsibility for the IZKS Mainz and follows its currently valid standard operation procedures (SOPs). The trial coordinator at the IZKS takes care of trial registration, trial center recruitment, study visit coordination and safety reports. The investigators at each trial center identify and recruit potential patients, obtain informed consent, and perform clinical examinations and documentation during the study visits. Support is provided by study nurses.

At two-week intervals, the study team meets digitally, and weekly meetings are held within the IZKS. Once per month, a short brief report and once per quarter, a report about reached study milestones and recruiting is sent to the G-BA.

### Composition of the data monitoring committee, its role and reporting structure {21a}

The Data Safety Monitoring Committee (DSMB) consists of two independent members with high expertise in traumatology and scientific methodology as well as one biometrician. The DSMB regularly evaluates, but at least every 12 months, the results of safety analyses, interim evaluations and their influence on the benefit-risk assessment for the study participants. The DSMB also assesses data validity and integrity as well as the feasibility of the trial after the recruitment of 15% of participants (~ *n* = 9). In addition, the DSMB can make recommendations for continuation, modification or even termination of the study. The entire responsibilities of the DSMB are fixed in the CAM-P-OS DSMB Charta, Version 1.0, April 17th, 2024.

### Adverse event reporting and harms {22}

All reported, detected or suspicious adverse effects will be recorded in terms of duration, frequency and severity in the database. Operative interventions or revision surgery will also be documented, although the removal of a set screw in cases of syndesmosis injury will not be considered an AE. All medical products used in this trial were applied for their purposes. Therefore, we assume that patient security is basically guaranteed by the manufacturers. If in doubt, all suspected incidents resulting from the use of the medical device will be documented in the database and reported to the manufacturer, the competent authority (Federal Institute for Drugs and Medical Devices) as well as to the responsible Ethics Committee. All the data are continuously analyzed and regularly presented to the DSMB.

### Frequency and plans for auditing trial conduct {23}

Clinical monitoring is carried out in accordance with the IZKS SOPs. The details and rationale of the monitoring strategies are specified in the monitoring plan. At defined intervals, study monitors visit the participating study centers to check accordance with the study protocol, GCP, and legal requirements. Additional digital remote visits by the monitors are implemented to check administrative aspects and plausibility.

### Plans for communicating important protocol amendments to relevant parties (e.g., trial participants, ethical committees) {25}

Neither the investigators nor the sponsor is allowed to change the study protocol without the written consent of the other party. Subsequent changes to the study protocol, changes to the investigators, changes in the members of the DSMB, changes to the study centers and changes in other documents must first be approved by the responsible ethics committee and the G-BA in Berlin.

### Dissemination plans {31a}

The complete results of this prospective trial will be published in international peer-reviewed journals and presented at national and international conferences.

## Discussion

The use of ACM in a closed kinetic chain seems to be beneficial in short-term rehabilitation protocols after anterior cruciate ligament repair and shows encouraging results in the midterm after surgically treated distal fibular fractures [6, 8]. Recently, a subgroup analysis in the systematic review of Chen et al. reported better functional scores and an earlier return-to-work/daily life in patients undergoing early weight bearing along with active movement 12 weeks after surgically treated ankle fractures [21]. Since fractures of the lower extremities lead to an average of 61.7 days of incapacity for work, this results in serious socioeconomic consequences [22]. Moreover, there is a lack of physiotherapy professionals in Germany [23], leading to long waiting times with a prolonged rehabilitation process [24]. The ACM device used in this study was developed to support and expand standard physiotherapy protocols. The purpose of the CAM-P-OS trial is to prove the superiority of an ACM device in a standardized aftercare protocol after open reduction and internal fixation of distal fibular fractures of Weber types B and C. Based on the results of this study, the G-BA is considering approving this procedure as part of a future rehabilitation protocol in Germany and allowing patients throughout the country to have an ACM device prescribed by their physician. In summary, it is hoped that this multicenter trial will provide valid data that will have a positive impact on the treatment of surgically treated, isolated fibula fractures in times of scarce resources and high socioeconomic pressure.

### Limitations

There are several limitations that need to be mentioned. First, patients randomized to the ACM device have higher training times than those in the control group. This could lead to better results on the one hand but also to a possibly inaccurate technique when the device is used, reduced compliance due to the training effort and possibly bad influence on wound healing on the other hand. We are aware of this limitation, but we wanted to prevent patients with ACM devices from not doing their own exercises, as these are more than just mobilization. Second, there is the potential risk of incorrect self-entries in the training diaries. Therefore, we implemented additional questionnaires for the physiotherapists involved to obtain objective information about patients’ adherence to the training. Third, we know that it is a risk to compare the effectiveness of the FAOS-ADL [9] with that of the VAS [16] during sample size planning. However, since there are no data using the FAOS-ADL in the comparison of an ACM device with standard treatment, we assume that the FAOS-ADL score is correlated with the VAS score.

### Trial status

The current protocol number is Version 1.1, June 6th, 2024. Recruitment started in October 2024. Completion is expected in April 2026.

## Data Availability

Depending on a reasonable request, collected trial data or their analysis can be made available.

## Abbreviations

ACM: Active controlled motion
AEs: Adverse events
AOFAS: American Orthopedic Foot and Ankle Society
CAM: Controlled active motion
DMP: Data Management Form
DSMB: Data Safety Management Board
eCRF: Electronic Case Report Form
FAOS: Foot and Ankle Outcome Score
FAOS-ADL: Foot and Ankle Outcome Score – Activities of Daily Living
G-BA: Gemeinsamer Bundesausschuss (German Joint Federal Committee)
GCP: Good clinical practice
ICD-10: International Statistical Classification of Diseases and Related Health Problems; Version 10
ICE: Intercurrent event
ICH: International Council for Harmonization of Technical Requirements for Registration of Pharmaceuticals for Human Use
ISF: Investigator site file
ITT: Intention-to-treat population
IZKS: Interdisziplinäres Zentrum für Klinische Studien (= Interdisciplinary Center for Clinical Trials)
NRS: Numeric Rating Scale
ORIF: Open reduction and internal fixation
ROM: Range of Movement
SF-36: Short Form 36 questionnaire
SOP: Standard operation procedure
VAS: Visual analog scale
